# Emergency presentation of colorectal patients in Spain

**DOI:** 10.1371/journal.pone.0203556

**Published:** 2018-10-01

**Authors:** Magdalena Esteva, Mercedes Ruiz-Díaz, M. Antonia Sánchez, Sonia Pértega, Salvador Pita-Fernández, Francesc Macià, Margarita Posso, Luis González-Luján, Marta M. Boscá-Wats, Alfonso Leiva, Joana Ripoll, On behalf of the DECCIRE GROUP

**Affiliations:** 1 Primary Care Research Unit, Majorca Department of Primary Care; Baleares Health Service [IbSalut], Balearic Islands Health Research Institute (IdISBa), Majorca, Spain; 2 Faculty of Health Sciences, University of Zaragoza, Zaragoza, Spain; 3 Centro de Salud Fuentes Norte Zaragoza, Department of Medicine, Psychiatry and Dermatology, University of Zaragoza, Zaragoza, Spain; 4 Clinical Epidemiology and Biostatistics Research Group, Instituto de Investigación Biomédica de A Coruña (INIBIC), Complexo Hospitalario Universitario de A Coruña (CHUAC), SERGAS, Universidade da Coruña, A Coruña, España; 5 Epidemiology and Evaluation Department, Hospital del Mar, Barcelona, Spain; 6 IMIM (Hospital del Mar Medical Research Institute), Barcelona, Spain; 7 REDISSEC (Health Services Research on Chronic Patients Network), Madrid, Spain; 8 Serreria II Primary Care Centre, Valencia Institute of Health, Valencia, Spain; 9 Digestive Medicine Unit, Hospital Clinic Universitari de València, València, Spain; University Hospital Llandough, UNITED KINGDOM

## Abstract

**Background:**

Colorectal cancer (CRC) is the leading cause of cancer deaths in Europe. Survival is poorer in patients admitted to hospitals through the emergency department than in electively admitted patients. Knowledge of factors associated with a cancer diagnosis through presentation at an emergency department may reduce the likelihood of an emergency diagnosis. This study evaluated factors influencing the diagnosis of CRC in the emergency department.

**Methods and findings:**

This is a cross-sectional study in 5 Spanish regions; subjects were incident cases of CRC diagnosed in 9 public hospitals, between 2006 and 2008. Data were obtained from patient interviews and primary care and hospital clinical records. We found that approximately 40% of CRC patients first contacted a hospital for CRC through an emergency service. Women were more likely than men to be emergency presenters. The type of symptom associated with emergency presentation differed between patients with colon cancer and those with rectal cancer, in that the frequency of “alarm symptoms” was significantly lower in colon than in rectal cancer patients who initially presented to emergency services. Soon after symptom onset, some patients went to a hospital emergency service, whereas others contacted their GP. Lack of contact with a GP for CRC-related symptoms was consistently related to emergency presentation. Among patients who contacted a GP, a higher number of consultations for CRC symptoms and any referral to outpatient consultations reduced the likelihood of emergency presentation. All diagnostic time intervals were shorter in emergency presenters than in elective patients.

**Conclusions:**

Emergency presenters are not a uniform category and can be divided into categories according to their symptoms, help seeking behavior trajectory and interaction with their GPs. Time constraints for testing and delays in obtaining outpatient appointments led patients to visit a hospital service either on their own or after referral by their GP.

## Introduction

Colorectal cancer (CRC) is one of the most frequent tumors and the leading cause of cancer deaths in Europe [1]. Although relative survival has increased since the 1980s, the 5-year survival rates in countries with the best survival rates are still only approximately 60% [2]. Additionally, survival is poorer in cancer patients admitted to hospitals through the emergency department than in electively admitted patients [3–6], even after adjusting for age and morbidity [7–8].

Clinical conditions are generally more complicated in patients diagnosed through an emergency service than in patients diagnosed through outpatient elective consultations. Patients diagnosed through an emergency service present with higher rates of complications, including obstructions [43%] [8], bowel perforation and peritonitis [9], and they more frequently undergo emergency surgery. These factors contribute to higher rates of preoperative mortality and postoperative morbidities [8–9], factors that contribute to poorer overall outcomes [10–11]. Thus, diagnosing cancers before a patient reaches a state requiring presentation at an emergency department may improve patient survival [12].

Knowledge of factors associated with a cancer diagnosis through presentation at an emergency department may reduce the likelihood of an emergency diagnosis. These factors may include personal and clinical characteristics, as well as the performance of health services before an emergency contact. In some patients, these factors may be related to tumor aggressiveness or anatomical location, whereas in other patients a lack of prior symptoms may prevent patients from contacting their general practitioners (GPs). Moreover, some patients who experience symptoms may not seek help promptly, or doctors may miss diagnostic opportunities because of atypical symptoms, barriers to referral or long hospital waiting lists [3, 13].

Regarding factors related to the route of diagnosis, a few studies have examined differences between colon and rectal cancers [14–17]. Because these cancers represent entities with different molecular, clinical, pathologic and biological characteristics, emergency presentation and the factors associated with emergency presentation should be assessed separately for patients with colon and rectal cancers [18]. The present study evaluated factors influencing the diagnosis of CRC in the emergency department, analyzing data for all CRC patients as well as colon and rectal cancer patients as separate groups.

## Methods

### Population and study setting

This multicenter, cross-sectional study involved patients in five regions of Spain (Aragón, Balearic Islands, Barcelona, Galicia and Valencia). Consecutive patients diagnosed with CRC (International Disease Classification 153–154) and registered with a GP were recruited through the pathology services of nine public hospitals between September 2006 and September 2008. Patients with prevalent or recurrent CRC or multiple tumors and patients diagnosed in private hospitals were excluded. Patients were contacted by their oncologist during the inpatient stage or during an outpatient oncology visit. Individual specialists invited these patients to participate in this study. All participants provided written informed consent. The methods used in this study have been published elsewhere [19–20].

### Data collection procedures

Data were obtained by specifically trained GPs and nurses from patient interviews, together with reviews of primary care and hospital records. Data obtained from patient interviews included socio-demographic factors, such as age, sex, marital status, and level of education, history of cancer in family members or acquaintances, and initial symptom/s: Each patient was asked how long he/she had been feeling unwell. Symptoms spontaneously mentioned by the patient were considered as the initial symptoms for that patient and the date was recorded. If the patient could remember the exact day, then that date was recorded. If the patient could not accurately remember the date of onset then the approximate date was recorded; for example, if a patient reported symptom onset two months earlier, the date recorded was two months before the date of the interview. After recording voluntary data on disease onset, the interviewer asked each patient if he/she presented with any of the other symptoms on a 22 symptoms checklist. To assess perception of symptom seriousness, the patient was asked if he/she considered the initial symptoms very serious, quite serious, not serious, or other. To assess help-seeking action, the patient was asked what he/she did after the onset of first symptoms: visit a doctor, wait for symptoms to clear, or other. The type of doctor contacted and confidence in their GP (0–10, with 10 considered maximum confidence) were also recorded. The patients were interviewed after a median of 47 days after the diagnosis (IQR = 88 days).

Hospital records were reviewed after the interview. After determining the date of diagnosis from the pathology report, the data manager reviewed the records to determine the first patient contact related to CRC symptoms. Date of diagnosis was determined from the first pathology report. The first hospital service that evaluated the patient was dichotomized to presentation at the emergency department (EP) or to outpatient services (OS) (surgery, gastroenterology, internal medicine or others).

Primary care records were reviewed after the interview and the review of hospital records in order to have the precise date of diagnosis. Primary care records were reviewed for 2 years prior to the date of diagnosis to determine the first GP contact for CRC symptoms. If patients did not contact their GPs for CRC symptoms, no other data were collected. If patients did contact their GPs, then the data recorded included the number of subsequent visits for CRC symptoms to GPs prior to diagnosis as well as the number of primary care visits to a GP or nurse during the 12 months before the diagnosis, and any suspicion of diagnosis registered in a patient’s clinical record. In addition, the Charlson Comorbidity Index at diagnosis was recorded from both hospital and primary care clinical records.

Symptom duration was divided into several intervals and calculated in days. 1) The patient interval was defined as the number of days from the onset of first CRC symptoms to first consultation with a physician; 2) the health services interval was defined as the time from first contact with a physician to diagnosis; 3) the diagnosis interval was defined as the time from the onset of first CRC symptoms to the date of diagnosis; and 4) the total interval was defined as the time from first presentation with CRC symptoms presentation to date of treatment. Patient interval and total interval were calculated according to the Aarhus Statement [21].

### Statistical analysis

Data are presented as numbers and percentages or as medians and interquartile ranges (IRs). Data on patients with colon and rectal cancer were examined separately throughout. Independent variables associated with the crude proportions of patients diagnosed through the EP or other hospital services were assessed by the Chi-Square test, whereas the association between differences in symptom duration and emergency presentation were analyzed using the Mann Whitney U-test. A P-value <0.05 was considered statistically significant.

Factors independently predictive of emergency presentation or of initial consultation with a GP or other doctor were analyzed by adjusted multivariate logistic regression analysis, as was GP performance among patients who consulted a GP. Any factor found by univariate analysis to be significant at P≤0.10 was included in the multivariate analysis. Interactions between factors included in the final models were examined. Sensitivity analyses of the models assessing emergency presentation and initial consultation were performed, without excluding the variables ‘first contact with health services’ and ‘hospital service of first referral’, respectively, as they could confound the associations of the other independent variables. All statistical analyses were performed using the Statistical Package for Social Sciences (SPSS version 23.0).

This study was approved by the Primary Health Care Committee of each health district and by the Ethical and Clinical Research Committee of each participating region (Comitè d'Ètica de la Investigació de les Illes Balears; Comité Ético de Investigación Clínica (CEIC) del Hospital Clínico Universitario de Valencia; Comité Ético de Investigación Clínica de Galicia; Comitè Ètic d'Investigació Clínica del Parc de Salut Mar; Comité Ético de Investigación Clínica de Aragón).

## Results

A total of 950 patients with CRC were included in this study. Of the pre-included patients 82 died before interview; 26 were excluded because we could not obtain informed consent; 56 were included as informed consent was obtained from their principal caregiver for clinical record’s data review. Of the 950 patients, 11 were excluded as there was no information on their initial presentation at a hospital. Of the 939 included patients, 794 (84.6%) were symptomatic, 84 (8.9%) were detected during screening, and 61 (6.5%) were incidentally diagnosed with CRC. In addition, 592 (63.0%) patients were diagnosed with colon cancer and 347 (37.0%) with rectal cancer. Sixty-eight (7.2%) patients did not respond to requests for interviews.

We found that 430 patients (45.8%), 300 with colon cancer (50.7%) and 130 with rectal cancer (37.5%), initially accessed hospital care through emergency services (P<0.001). Of these 430 patients, 363 (84.4%) were symptomatic, 43 (10.0%) were detected during screening, and 24 (5.6%) were detected incidentally. By comparison, of the 559 patients who initially presented to other hospital services, 78.8% were symptomatic, 12% were detected during screening, and 9.2% were detected incidentally (P = 0.53).

Table 1 presents the socio-demographic characteristics of the patients in this study. Among patients with colon cancer, the frequency of emergency presentation was higher in older than in younger patients and higher in single or widow/separated/divorced patients than in married patients, but these differences were not significant. Among patients with rectal cancer, the frequency of emergency presentation was higher in women than in men. In both cohorts, the frequency of emergency presentation was lower in patients who did than did not have family members or acquaintances with cancer, but the difference in colon cancer patients was not statistically significant.

**Table 1:** Socio-demographic characteristics of patients relative to tumor location and access to hospital (patient interviews).

	Global N = 939		P value	Colon n = 592		P value	Rectum n = 347		P value
	EP n (%)	OS n (%)		EP n (%)	OS n (%)		EP n (%)	OS n (%)	
**Age, years**			0.23			0.06			0.93
**<**50	24 [5.7]	31 [6.2]		16 [5.4]	19 [6.7]		8 [6.3]	12 [5.7]	
50–64	95 [22.5]	122 [24.5]		64 [21.6]	65 [22.8]		31 [24.6]	57 [26.9]	
65–74	121 [28.7]	154 [31.0]		81 [27.4]	94 [33]		40 [31.7]	60 [28.3]	
75–84	142 [33.6]	162 [32.6]		101 [34.1]	92 [32.3]		41 [32.5]	70 [33.0]	
≥85	40 [9.5]	28 [5.6]		34 [11.5]	15 [5.3]		6 [4.8]	13 [6.1]	
**Sex**			0.09			0.89			0.02
Male	251 [59.6]	325 [65.0]		174 [59.4]	172 [59.9]		77 [60.2]	153 [71.8]	
Female	170 [40.4]	175 [35.0]		119 [40.6]	115 [40.1]		51 [39.8]	60 [28.2]	
**Marital Status**			0.14			0.07			0.87
Single	35 [8.9]	31 [6.5]		29 [10.5]	22 [8.1]		6 [5.1]	9 [4.5]	
Married	269 [68.4]	353 [74.3]		179 [65.1]	202 [74.0]		90 [76.3]	151 [74.8]	
Widow/Separated/Divorced	89 [22.6]	91 [19.2]		67 [24.4]	49 [17.9]		22 [18.6]	42 [20.8]	
**Level of education**			0.29			0.17			0.68
Illiterate-incomplete primary	55 [14.0]	66 [13.9]		34 [12.3]	37 [13.6]		21 [17.9]	29 [14.3]	
Primary education	199 [50.6]	257 [54.0]		138 [50.0]	146 [53.5]		61 [52.1]	111 [54.7]	
Secondary education	77 [19.6]	71 [14.9]		61 [22.1]	39 [14.3]		16 [13.7]	32 [15.8]	
High school	41 [10.4]	46 [9.7]		27 [9.8]	28 [10.3]		14 [12.0]	18 [8.9]	
University education	231 [5.3]	36 [7.6]		16 [5.8]	23 [8.4]		5 [4.3]	13 [6.4]	
**History of cancer in family members and/or acquaintances’**			0.002			0.09			0.004
Yes	186 [47.2]	275 [57.9]		133 [48.2]	151 [55.3]		53 [44.9]	124 [61.4]	
No	208 [52.8]	200 [42.1]		143 [51.8]	122 [44.7]		65 [55.1]	78 [38.6]	
**Charlson Index Score**									0.38
Mean [SD]	1.11 [1.33]	1.00 [1.45]	0.25	1.13 [1.30]	1.07 [1.43]	0.56	1.05 [1.40]	0.91 [1.47]	

Table 2 shows the distribution of first symptoms, patient appraisal of symptoms, and help-seeking behavior and their relationship with emergency presentation. A higher number of initial symptoms were associated with emergency presentation in patients with colon cancer, but not in patients with rectal cancer. Among patients with colon cancer, emergency presentation was associated with abdominal pain, constipation, vomiting, and loss of weight, anorexia, and fatigue. Conversely, rectal bleeding was related with a lower probability of emergency presentation. Among rectal cancer patients, emergency presentation was significantly associated with constipation, vomiting and changes in bowel habits. Abdominal occlusion was present in one-third of patients with colon cancer and one-tenth of those with rectal cancer. Emergency presentation was unrelated to symptom severity appraisal and to help-seeking behavior, whether visiting a doctor after symptom onset or waiting for symptoms to clear. Under all situations, contact with a GP after symptom onset diminished the probability of emergency presentation as well being associated with a high score of patient confidence in their GPs.

**Table 2:** Initial patient symptoms, initial presentation and comorbidities relative to tumor location and access to hospital (patient interviews).

Variables	Global N = 939		P value	Colon n = 592		P value	Rectum n = 347		P Value
	EP n [%]	OS n [%]		EP n [%]	OS n [%]		EP n [%]	OS n [%]	
**Number of initial symptoms**			<0.001			<0.001			0.32
0	20 [5.1]	52 [11.1]		17 [6.2]	40 [14.9]		3 [2.6]	12 [6.0]	
1	182 [46.7]	256 [54.6]		124 [45.3]	146 [54.3]		58 [50.0]	110 [55.0]	
2–3	135 [34.6]	122 [26.0]		94 [34.3]	62 [23.0]		41 [35.3]	60 [30.0]	
>4	53 [13.6]	39 [8.3]		39 [14.2]	21 [7.8]		14 [12.1]	18 [9.0]	
First symptom*									
Abdominal pain [yes]	130 [33.3]	91 [19.4]	<0.001	110 [40.1]	66 [24.5]	0.001	20 [17.2]	25 [12.5]	0.24
Constipation [yes]	83 [21.3]	48 [10.2]	<0.001	56 [20.4]	32 [11.9]	0.007	27 [23.3]	16 [8.0]	<0.001
Rectal bleeding [yes]	104 [26.7]	164 [35.0]	0.009	49 [17.9]	72 [26.8]	0.01	55 [47.4]	92 [46.0]	0.80
Diarrhea [yes]	61 [15.6]	64 [13.6]	0.40	34 [12.4]	27 [10.0]	0.38	27 [23.3]	37 [18.5]	0.30
Changes in bowel habits [yes]	149 [38.2]	150 [32.0]	0.057	91 [33.2]	73 [27.1]	0.12	58 [50.0]	77 [38.5]	0.04
Vomiting [yes]	23 [5.9]	7 [1.5]	<0.001	19 [6.9]	6 [2.2]	0.009	4 [3.4]	1 [0.5]	0.04
Loss of appetite [yes]	40 [10.3]	31 [6.6]	0.053	33 [12.0]	19 [7.1]	0.049	7 [6.0]	12 [6.0]	0.99
Loss of weight [yes]	49 [12.6]	31 [6.6]	0.003	41 [15.0]	16 [5.9]	0.001	8 [6.9]	15 [7.5]	0.79
Fatigue [yes]	71 [18.2]	60 [12.8]	0.028	57 [20.8]	46 [17.1]	0.27	14 [12.1]	14 [7.0]	0.12
Anemia [yes]	38 [9.7]	38 [8.1]	0.39	31 [11.3]	27 [10.0]	0.63	7 [6.0]	11 [5.5]	0.88
Perception of symptoms*			0.74			0.45			0.79
Non-serious	237 [64.6]	275 [64.4]		157 [62.1]	147 [63.1]		80 [70.2]	128 [66.0]	
Serious-Very serious	109 [29.7]	130 [30.5]		79 [30.2]	74 [31.8]		30 [26.3]	56 [28.8]	
Others	21 [5.7]	22 [5.2]		17 [6.7]	12 [5.2]		4 [3.5]	10 [5.2]	
Intestinal occlusion*			<0.001			<0.001			0.02
Yes	101 [23.8]	26 [5.2]		88 [29.6]	18 [6.3]		13 [10.2]	8 [3.8]	
No	323 [76.2]	471 [94.8]		209 [70.4]	268 [93.7]		114 [89.8]	203 [96.2]	
Help seeking behavior*			0.40			0.44			0.81
Visit a doctor	257 [69.5]	312 [73.1]		180 [70.3]	175 [75.1]		77 [67.5]	137 [70.6]	
Wait to clear up	90 [24.3]	96 [22.5]		59 [23.0]	47 [20.2]		31 [27.2]	49 [25.3]	
Others	23 [6.2]	19 [4.4]		17 [6.6]	11 [4.7]		6 [5.3]	8 [4.1]	
Health services first contact*			<0.001			<0.001			<0.001
General practitioner	266 [70.6]	356 [81.7]		178 [68.2]	197 [81.7]		88 [75.8]	159 [81.5]	
Hospital emergency department	93 [24.7]	27 [6.1]		68 [26.1]	13 [5.4]		25 [21.6]	14 [7.2]	
Other	18 [4.8]	53 [12.2]		15 [5.7]	31 [12.9]		3 [2.6]	22 [11.3]	
**Confidence in their GP [0–10]**			0.39			0.049			0.29
0–4	32 [8.6]	29 [6.3]		25 [9.6]	11 [4.2]		7 [6.4]	18 [9.2]	
5–6	33 [8.9]	37 [8.1]		23 [8.8]	27 [10.3]		10 [9.1]	10 [5.1]	
7–10	306 [82.5]	392 [85.6]		213 [81.6]	224 [85.5]		93 [84.5]	168 [85.7]	

*only symptomatic. Global n = 794, colon cancer n = 486, rectal cancer n = 308

We also observed that socio-demographic characteristics and symptom appraisal were similar in patients who contacted an emergency service and those who contacted their GP or another doctor after onset of symptoms. However, abdominal pain and vomiting were significantly higher in emergency presenters (data not shown).

Of the 939 patients, 628 (66.9%) contacted their GP due to CRC symptoms. Table 3 shows patient-assessed performance of their GPs and its relationship with emergency presentation. After the first contact with a GP, the proportion of patients with more than three subsequent visits was lower in emergency than in non-emergency presenters. However, the two groups did not differ in the number of contacts with GPs and nurses for any problem during the previous 12 months. We also found that 48.4% of emergency presenters were referred to an emergency service by their GPs, during the course of diagnosis; 67.6% has had a test or investigation requested. From these patients, 33.8% has had at least one image investigation and 54.1% at least a test (blood test, and/or fecal occult test). We also found that 36.3% of emergency presenters were referred to outpatient’s services during the diagnostic process, compared with 76% of electively presenting patients. The likelihood of emergency presentation was significantly lower in all patients and in colon cancer patients, but not in rectal cancer patients, who attended teaching health centers.

**Table 3:** Performance of general practitioners relative to tumor location and emergency presentation to a hospital in patients who contacted a general practitioner for symptoms of colorectal cancer.

	Global N = 628		P value	Colon n = 382		P value	Rectum n = 246		P value
	EP n [%]	OS n [%]		EP n [%]	OS n [%]		EP n [%]	OS n [%]	
**Number of successive visits due to CRC symptoms**			0.002			0.005			0.07
0	80 [30.1]	94 [26.0]		54 [29.7]	42 [21.0]		26 [31.0]	52 [66.7]	
1–2	119 [44.7]	126 [34.8]		77 [42.3]	68 [34.0]		42 [50.0]	58 [35.8]	
3–5	51 [19.2]	96 [26.5]		38 [20.9]	61 [30.5]		13 [15.5]	35 [21.6]	
6+	16 [6.0]	46 [12.7]		13 [7.1]	29 [14.5]		3 [3.6]	17 [10.5]	
**Visits to primary care during the 12 months before CRC diagnosis**			0.67			0.55			0.56
0	3 [1.1]	4 [1.1]		3 [1.6]	3 [1.5]		0 [0.0]	1 [0.6]	
1–5	74 [27.8]	87 [24.0]		52 [28.6]	43 [21.5]		22 [26.2]	44 [27.2]	
6–12	93 [35.0]	120 [33.1]		56 [30.8]	62 [31.0]		37 [44.0]	58 [35.8]	
13–24	64 [24.1]	98 [27.1]		45 [24.7]	59 [29.5]		19 [22.6]	39 [24.1]	
> = 25	32 [12.0]	53 [14.6]		26 [14.3]	33 [16.5]		6 [7.1]	20 [12.3]	
**Suspicion of CRC in clinical records**			0.33			0.22			0.46
Yes	164 [62.1]	208 [58.3]		105 [58.3]	103 [52.3]		59 [70.2]	105 [65.6]	
No	100 [37.9]	149 [41.7]		75 [41.7]	94 [55.6]		25 [29.8]	55 [34.4]	
**Outpatient department first referral **			<0.001			<0.001			<0.001
Gastroenterology	60 [39.2]	197 [72.7]		38 [29.2]	92 [70.8]		22 [40.7]	105 [78.9]	
Surgery	7 [4.6]	32 [11.8]		2 [13.3]	13 [86.7]		5 [9.3]	19 [14.3]	
Hospital emergency department	74 [48.4]	23 [8.5]		50 [72.5]	19 [27.5]		24 [44.4]	4 [3.0]	
Others	12 [7.8]	19 [7.0]		9 [9.1]	14 [10.1]		3 [5.6]	5 [3.8]	
**Any referral to outpatient services**			<0.001			<0.001			<0.001
Yes	94 [36.3]	276 [76.0]		60 [34.1]	135 [67.5]		34 [41.5]	140 [87.5]	
No	165 [63.7]	87 [24.0]		116 [65.9]	65 [32.5]		48 [58.5]	20 [12.5]	
**Health center accreditation for teaching GPs**			0.005			<0.01			0.11
Teaching health center	62 [26.6]	126 [36.7]		43 [26.2]	73 [38.6]		19 [24.4]	53 [34.4]	
Non-teaching health center	180 [74.4]	217 [63.5]		121 [73.8]	116 [61.4]		59 [75.6]	101 [63.3]	

Multivariate analysis showed that the number of symptoms and abdominal occlusion were associated with emergency presentation in all patients and in those with colon cancer (Table 4). Rectal bleeding was associated with a lower probability of emergency presentation in colon cancer patients, whereas constipation was associated with a higher probability of emergency presentation in rectal cancer patients. Patient decision to consult a GP was inversely related to emergency presentation in all patients and in those with colon cancer. Among patients who contacted a GP, a higher number of subsequent visits were associated with a lower probability of emergency presentation and referral to an outpatient service.

**Table 4:** Multivariate logistic regression analysis of factors associated with colorectal cancer diagnosis.

**Variables based on patients interviews**						
** Variables**	**GLOBAL** **OR [95%CI]**	**P** **value**	**COLON** **OR [95%CI]**	**P** **value**	**RECTUM** **OR [95%CI]**	**P** **value**
**Sex**						0.01
Men					1	
Women					1.93 (1.11–3.18)	
**History of cancer in family members and/or acquaintances’**		0.09		-		0.053
No	1				1	
Yes	10,75 [0.54–1.04]				10,58 (0.34–1.006)	
**Number of initial symptoms**					-	-
1	1		1			
2–3	1.59 [1.11–2.27]	0.01	1.88 [1.19–2.98]	0.007		
> = 4	2.12 [1.14–3.96]	0.01	3.45 [1.50–7.92]	0.04		
**Rectal bleeding**		0.06		0.04	-	-
No	1		1			
Yes	0.71 [0.50–1.01]		0.60 [0.36–0.98]			
**Constipation**	-	-	-	-		0.001
No					1	
Yes					3.44 [1.61–7.36]	
**Health services first contact**		0.002		0.002	-	-
Other	1		1			
General practitioner	0.54 [0.36–0.80)		0.45 [0.27–0.75]			
**Intestinal occlusion**		<0.001		<0.001	-	-
No	1		1			
Yes	4.91 [2.72–8.87]		4,59 [2.37–8.91]			
Variables of GP performance in patients who contacted for CRC symptoms						
**Subsequent visits to GP for CRC symptoms**						
0	1		1			
1–2	1.31 [0.82–2.08]	0.25	1.04 [0.56–1.91]	0.90		
3–5	0.72 [0.42–1.21]	0.22	0.52 [0.27–1.14]	0.05		
≥6	0.49 [0.23–1.03]	0.06	0.40 [0.16–0.97]	0.04		
**Any referral to outpatient services**		<0.01		<0.01		<0.01
No	1		1		1	
Yes	0.18 [0.12–0.27]		0.25 (0.15–0.39)		0.11 [0.06–0.22]	

Table 5 shows the relationships of four diagnostic time intervals (patient interval, health services interval, diagnosis interval, total interval) with emergency presentation. All time intervals were lower in patients who used emergency services during their first contact with a hospital.

**Table 5:** Diagnosis intervals according to emergency presentation.

	Global N = 939		P value	Colon n = 592		P value	Rectum n = 347		P value
	EP Median (P25-P75)	OS Median (P25-P75)		EP Median (P25-P75)	OS Median (P25-P75)		EP Median (P25-P75)	OS Median (P25-P75)	
**Patient interval**	14 [2,0–61.0]	30 [5.0–92.0]	<0.001	13 [2.0–59.5]	30 [5.0–101.5]	0.005	14 [2.0–61.0]	30 [5.0–92.0]	0.00
**Health services interval**	39 [15.0–111.0]	84 [38.5–200.0]	<0.001	41 [16.5–106.7]	79 [38.5–169.5]	<0.001	39 [15.0–11.0]	84 [35.5–200.0]	<0.001
**Diagnosis interval**	86 [33.0–185.0]	163 [82.5–311.5]	<0.001	86.5 [32.7–186.7]	153 [86.5–307.0]	<0.001	86.0 [33.0–185.0]	163 [86.0–311.5]	<0.001
**Total interval**	106.0 [51.5–208.5]	195 [118.0–353.0]	<0.001	115,0 [60.7–214.5]	197 [118.5–363.5]	<0.001	106,0 [51.5–208.5]	195 [118.0–353.0]	<0.001

IQ = interquartile range

## Discussion

To our knowledge, few studies to date have assessed the factors associated with emergency presentation of patients with colon and rectal cancers. We found that in Spain approximately 40% of these patients first contacted a hospital for CRC through an emergency service, a higher percentage than in other countries. We also found that the type of symptom associated with emergency presentation differed between patients with colon cancer and those with rectal cancer, in that the frequency of “alarm symptoms” was significantly lower in colon than in rectal cancer patients who initially presented to emergency services. In both cohorts, however, the probability of emergency presentation increased with an increasing number of symptoms. Emergency presenters are not a uniform category and can be divided into categories according to their help seeking behavior trajectory and interaction with their GPs. Soon after symptom onset, some patients went to a hospital emergency service, whereas others contacted their GP. One of the most important factors associated with emergency presentation was a lack of contact with a GP for CRC-related symptoms. All diagnostic time intervals were shorter in emergency presenters than in elective patients.

### Strengths and limitations

One of the strengths of the study was our inclusion of important patient-reported data, providing insight into patient perception of symptoms and help-seeking behavior after symptom presentation. In addition, our inclusion of medical records compiled by GPs enabled our inclusion of important data reported by patients and considered relevant by their doctors.

Most of the limitations of this study have been described elsewhere [20–21]. First, there is no universally accepted definition of an emergency diagnosis of cancer. This constitutes the cornerstone for obtaining robust and comparable measures of different routes of diagnosis. As in most studies using routine data, some of these data were incomplete, limiting our ability to examine certain key characteristics and the confidence of our conclusions. Our findings may have been affected by missing data for some variables, mainly those from GP clinical records. Inclusion of patients diagnosed by screening or incidentally may also have biased our results. We found that a small proportion of these patients contacted a hospital through emergency services, perhaps because a GP or gastroenterologist referred these patients to emergencies based on the results of endoscopy or after an incidental detection of cancer in order to overcome waiting lists. Patient and GP recall of first symptoms did not always align, particularly for vague symptoms [22], and there were differences in recalling the date of first symptom presentation. Furthermore, the percentage of patients reported visiting a GP after symptom onset was higher than that reported in their primary care records, indicating that patients may have overestimated their contact with a GP or that some GPs may not have recorded vague symptoms.

### Comparison with other studies and interpretation of our findings

The percentage of CRC patients who initially presented to emergency services was much higher in our study than in studies performed in other European and North America countries, which have been reported to range from 17–26% [6; 23–26]. One explanation for these differences may be differences in the definition of emergency presentation. Although several studies have used an algorithm to define emergency presentation [27], other studies regard emergency presentation as visiting an emergency service once before the diagnosis of CRC [27] or have used other criteria [25–26]. Our study defined emergency presentation as first patient contact with a hospital through an emergency service. Alternatively, GPs in Spain frequently refer patients with a high suspicion of cancer to emergency services to overcome limited access to investigations and outpatient appointments.

We found that the socio-demographic characteristics of these patients generally did not affect the rate of emergency presentation, although women with rectal, but not colon, cancer were more likely to use this route. Other studies have reported socio-demographic differences in emergency presentation, particularly in more vulnerable groups, including women, older patients, patients of low socioeconomic status and patients with higher comorbidity rates [17, 28–31]. These more vulnerable populations have less access to health services and usual care [32] and to colorectal examinations [33]. The discrepancy between our study and these other studies may be explained by the equal geographic distribution of primary care centers and hospitals in Spain. All groups would therefore have equal access to diagnostic examinations, specialists, and outpatient resources.

As shown in other studies, we observed that the type of initial symptom was highly associated with emergency presentation. Emergency presenters are more likely to present with abdominal pain [8,16,25,34], constipation [24,16,34], loss of weight [3, 34] fatigue and vomiting and significantly less likely to present with rectal bleeding [16,25,34,35] or changes in bowel habits [35]. Abdominal pain and abdominal obstruction are much more common in colon than in rectal cancer patients [8, 30], which may explain the higher rate of emergency presentation in those with colon cancer [3; 16, 17, 29]. Moreover, emergency presenters have symptoms of low predictive value, that is, not NICE-qualifying symptoms [25, 30, 36], and a higher number of symptoms. These findings suggest the need to review the role of symptoms with low predictive value for cancer when they appear simultaneously with other symptoms. However, emergency presenters do not report higher symptom severity, with similar proportions of emergency and non-emergency presenters contacting a doctor and disclosing symptoms to family and friends. Escalating or persisting symptoms, rather than severity of symptoms, could influence their visits to emergency services as described by others [37].

As previously reported [38], patients diagnosed with CRC by emergency services are quite heterogeneous. Some patients who experience symptoms go directly to an emergency service, whereas others visit their GP. The former group likely includes patients who postpone consultation for their symptoms, as well as those who respond immediately after experiencing disruptive symptoms, such as vomiting and abdominal pain [38].

Among patients who decided to visit a GP after symptom onset in the present work, fewer presented to emergency services, in agreement with other studies [25, 29–30]. Previous studies have found that, although similar proportions of emergency and non-emergency presenters consulted a primary-care physician [16, 25, 27], lower percentages of the former had ≥3 successive visits (25.2% vs. 39.2%) and referrals to outpatient consultations (36.3% vs. 76.0%). In addition, a higher proportion of emergency presenters were initially referred to an emergency service. These findings indicate that the diagnostic process in emergency presenters included fewer consultations and more referrals to emergency services by their GPs. Although it could be partially explained by the time from the clinical onset to diagnosis in these patients is inherently shorter and therefore the probability of having more consultations or referrals could be lower. Emergency presenters may have had more symptoms or more disruptive ones or may have been referred to emergency services to bypass long waiting lists or. Additionally some patients referred to an outpatient service may have contacted the hospital through an emergency service. Similarly, 30% of CRC patients in Scotland appropriately referred to secondary care were found to have been admitted to emergency services in the period between their referral and the appointment date [26].

Although emergency presentation has been reported to be a proxy of a delay in diagnosis [25], we found that the different intervals in the diagnostic pathway were shorter in emergency than in non-emergency presenters. These differences in symptom duration intervals may have been due to emergency presenters having more aggressive tumors, leading patients to quickly seek help and doctors to accelerate the testing schedules to arrive at a more rapid diagnosis. This may partly explain the waiting time paradox, in that patients with shorter times to diagnosis had higher mortality rates [39]. This does not mean that emergency admissions should be ignored but that the potential mortality benefits from a reduction may be less than hoped.

Finally, we found that patients registered in a teaching center were less likely to become emergency presenters than patients in non-teaching health services. Teaching health centers are characterized by better quality performance indicators and higher levels of educational activities, which may result in fewer referrals to emergency services. These findings are in agreement with a study showing that a higher total quality and outcomes framework protected against unplanned admissions [23].

## Conclusions

In conclusion, our study indicates that emergency presentation is complex and does not have a single cause. Women were more likely than men to be emergency presenters. Specific symptom patterns differed in emergency and non-emergency presenters and in patients with colon and rectal tumors. Emergency presenters had a higher frequency of symptoms not included on the NICE qualifying list and a higher number of symptoms, but symptom severity was similar in emergency and non-emergency presenters. After their first symptoms, some patients opted not to contact a GP, with this being the most important predictor of emergency presentation. Among patients who contacted a GP, a higher number of consultations for CRC symptoms and any referral to outpatient consultations reduced the likelihood of emergency presentation. Time constraints for testing and delays in obtaining outpatient appointments led patients to visit a hospital service either on their own or after referral by their GP. Future research targeting different categories of emergency presenters is needed to identify the reasons for emergency presentation and to formulate interventions to prevent emergency presentation.
