# Correction: Emergency presentation of colorectal patients in Spain

**DOI:** 10.1371/journal.pone.0207143

**Published:** 2018-11-02

**Authors:** Magdalena Esteva, Mercedes Ruidíaz, M. Antonia Sánchez, Sonia Pértega, Salvador Pita-Fernández, Francesc Macià, Margarita Posso, Luis González-Luján, Marta M. Boscá-Wats, Alfonso Leiva, Joana Ripoll, On behalf of the DECCIRE GROUP

The second author’s name is spelled incorrectly. The correct name is: Mercedes Ruidíaz. The correct citation is: Esteva M, Ruidíaz M, Sánchez MA, Pértega S, Pita-Fernández S, Macià F, et al. (2018) Emergency presentation of colorectal patients in Spain. PLoS ONE 13(10): e0203556. https://doi.org/10.1371/journal.pone.0203556
